# Bladder Urothelial Carcinoma in a Child: Case Report and Review of Literature

**DOI:** 10.3389/fped.2019.00385

**Published:** 2019-09-20

**Authors:** Marian Hanae Oda, Danilo Vicente dos Santos, Adria Karina Farias, Leilane de Oliveira, Bruno Pinheiro Falcão, Nicholas J. Ahn, Antônio Carlos Amarante, Graziele Moraes Losso, Andre Ivan Bradley dos Santos Dias, Miguel Angelo Agulham, Camila Girardi Fachin

**Affiliations:** ^1^Medical School, Federal University of Parana, Curitiba, Brazil; ^2^Pediatric Surgery Department, University Hospital, Federal University of Parana, Curitiba, Brazil; ^3^The Children's Hospital of Philadelphia, Philadelphia, PA, United States; ^4^Mantis Diagnósticos Avançados, Curitiba, Brazil

**Keywords:** urothelial carcinoma, macroscopic hematuria, children, BRAF, KRAS

## Abstract

Bladder urothelial carcinoma (UC) it is the fifth most prevalent carcinoma in humans, nevertheless in children and young adults it's very rare. It usually occurs in older adults. Literature on UC in pediatric population is limited and important information (risk factors, follow-up protocols, etc.) are poorly defined. We present an 11-year-old boy with a painful macroscopic hematuria. Ultrasound revealed a heterogeneous intravesical mass without extravesical extension, which was confirmed by computed tomography (CT) and magnetic resonance imaging (MRI). The first biopsy was compatible with urothelial papilloma. After 1 year, he returned with a bigger mass. Transurethral resection of the bladder (TURB) was performed and immunohistochemistry showed low-grade papillary UC with a high-grade component, with tumor free margin. Tumor had mutations in the BRAF and KRAS genes. Two and a half years after the resection the patient has no recurrence. Less than 1% of bladder UC occur in the first two decades of life. Gross hematuria is a common symptom. Ultrasound is generally the first diagnostic tool. MRI is also helpful, but cystoscopy allows definitive diagnosis. Transurethral resection of the bladder (TURB) is the standard treatment, with good results and low recurrence rate, and it was the treatment of choice for our patient, that remains free of disease. The BRAF and KRAS gene mutations were never described before in pediatric UC. There are only few cases in literature of pediatric UC that present a tumor genetic profile; therefore, our case report adds more information to this very rare disease in children.

## Background

Urothelial carcinoma (UC) of the bladder typically occurs in patients in their sixth or seventh decade of life. It's a rare entity in children and young adults. Reportedly, they occur in 1–2.4% of the population younger than 40 years, however only in 0.1–0.4% in the first two decades of life (1–3).

Particularly, urothelial carcinoma accounts for 2.1% of all cancer related deaths. It affects both genders, with a male-to female ratio of 7:1, based on adult literature (4). However, the literature is very limited regarding the pediatric population with only about 100 cases reported from 1950 to 2013 (5, 6).

There is significant debate regarding the prognostic value of young age at diagnosis, with conflicting reported on literature (4). In bladder UC, tumors have been described as having a low grade of malignancy and with little tendency to recur (3, 5, 7). The histopathology of low grade UC is characterized by it's orderly appearance and variation in architectural and/or cytological features (4). However, the rarity of UC in children makes conclusions related to etiology, invasive potential, treatment, and surveillance difficult (6). The aim of this study is to report a case of urothelial carcinoma of the bladder in an 11-year-old patient, highlighting the clinical presentation, diagnosis, treatment, follow up, and tumor genetic profile.

## Materials and Methods

A retrospective review of the medical records was performed. Written informed consent was obtained from the parents and the patient for the publication of this case report, including the use of images and other relevant information. The study was approved by the Human Research Ethics Committee of the University Hospital. All subjects gave written informed consent in accordance with the Declaration of Helsinki.

For molecular analysis, DNA was extracted from the paraffin block in an automated way (QIACube). The Next Generation Sequencing (NGS) was performed on Gene Reader platform (QIAGEN) for regions and variants of clinical interest of the genes: KRAS, NRAS, KIT, BRAF, PDGFRA, ALK, EGFR, ERBB2, PIK3CA, ERBB3, ESR1, and RAF1. The result was coverage of 100% of bases with depth above 200×. The readings were aligned against the UCSC reference genome (hg19) and processed in Clinical Insight Analyze (QCI-A) software. The variants found were classified as Pathogenic, Probably Pathogenic, Benign, Probably Benign, and Uncertain Significance Variants (USV), according to the criteria of American College of Medical Genetics.

## Case Report

**Figure 1:**
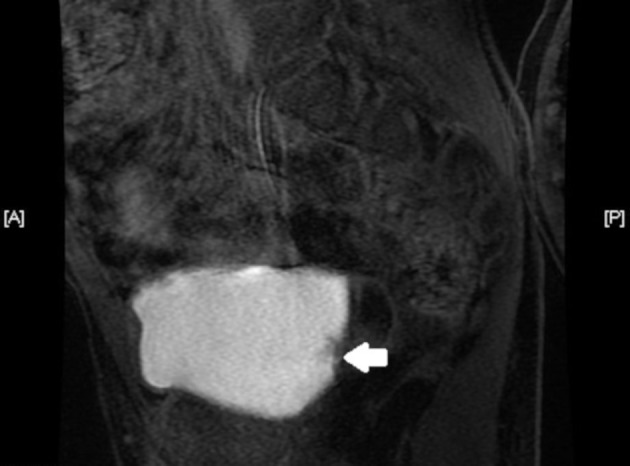
Abdominal MRI, in axial section, showing the lesion (indicated by the white arrow) restricted to the bladder mucosa, close to the right ureteral ostium.

An 11-year-old male presented at the University Hospital with painful macroscopic hematuria for 3 months. A urinary tract ultrasound showed a heterogeneous intravesical mass measuring 23 × 21 mm with papillary projections on the surface. Abdominal and pelvic CT scans with contrast showed a 20 mm mass in the right posterolateral bladder wall. The CT scan was unable to determine whether there was tumor extravesical extension, so the patient underwent an MRI with contrast that showed that tumor was restricted to the bladder mucosa (Figure 1). There was no history of passive smoking or industrial exposure due to proximity of residence.

**Figure 2:**
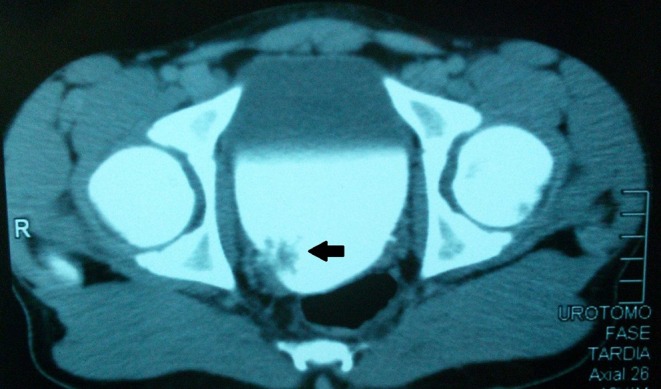
Abdominal CT scan, in axial section, showing the lesion (indicated by the black arrow) in the right posterolateral bladder wall.

**Figure 3:**
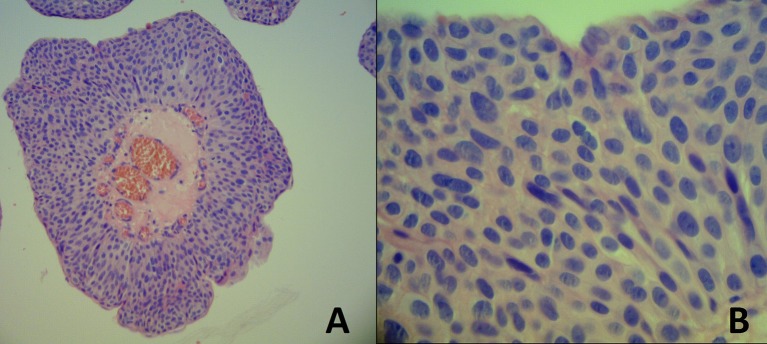
**(A)** Papillary urothelial carcinoma with some degree of cytoarchitectural disorder with moderate atypia. HEx100. **(B)** Detail of an area of high-grade atypia. HEx400.

Cystoscopy demonstrated a vegetative lesion in the right lateral bladder wall. Incisional biopsy was performed and histopathology revealed urothelial papilloma. Subsequently the patient was lost to follow-up and returned after 1 year with sporadic painless hematuria. A new CT scan with contrast identified a 29 × 22 mm solitary lesion in the same topography (Figure 2). The patient underwent a cystoscopic transurethral resection of the lesion (TURB). The histopathological exam was compatible with atypical papillary urothelial hyperplasia, but immunohistochemistry favored the diagnosis of low-grade papillary urothelial carcinoma, with high-grade component and without invasion to the subepithelial tissue and muscularis propria, with free margins (Figures 3A,B). The specimen was positive for p53 mutation on immunohistochemistry.

On follow-up, ultrasound was performed every 3 months and cystoscopy every 6 months. Two and a half years after the resection, the patient is cancer free.

The tumor genetic profile presented a pathogenic variant in the BRAF gene (c.1894C> T p.P632S), a probably pathogenic variant KRAS (c.97G> A p.D33N), and nine variants of uncertain significance in the ALK, EGFR, ERBB2, ERBB3, KIT, and PIK3CA (Table 1).

**Table 1:** Tumor genetic profile.

**Gene**	**Allele fraction**	**Function**	**Pathogenicity**
BRAF	c.1894C>T p.P632S	4.30% loss	Likely pathogenic
ALK	c.3451-23C>T	4.04% normal	Uncertain significance
EGFR	c.2184+27G>A	18% normal	Uncertain significance
ERBB2	c.3527G>A p.G1176E	4.15% normal	Uncertain significance
ERBB3	c.947G>A p.G316E	4.11% loss	Uncertain significance
ERBB3	c.875-37C>T	4.28% normal	Uncertain significance
KIT	c.1549C>T p.H517Y	5.10% normal	Uncertain significance
KRAS	c.97G>A p.D33N	4.14% loss	Probably pathogenic
PIK3CA	c.523C>T p.P175S	4.36% normal	Uncertain significance
PIK3CA	c.-77+8441G>A	8.86% normal	Uncertain significance
PIK3CA	c.353-65delA	4.43% normal	Uncertain significance

## Discussion

We reported a case of an 11-year-old male patient with UC of the bladder. This type of cancer is very rare in children and young adults. However, despite the low frequency of cases [only 0.1–0.4% of UC occur in the first two decades of life (1–3)], bladder UC were the predominant bladder cancer occurring in patients between 13 and 18 years (2).

Regarding clinical features, authors describe the presence of gross hematuria in 80% of published cases (6). Our patient presented only with painful macroscopic hematuria, although painless hematuria is more common (3). A wide range of other unusual presenting symptoms were reported in literature [urinary frequency, recurrent cystitis, pyelonephritis, obstruction, abdominal pain, flank pain, fever, hematospermia, nephrolithiasis, and emesis (8)]. Incidental bladder mass finding has also been describe (9).

Risk factors for TCC among the pediatric population are poorly defined. Fifty percent of TCC in adults is attributed to tobacco use (8). In pediatrics, one study reports a 16-years old boy who was a steady smoker (5). A case series revealed three cases of urothelial carcinoma in children with a history of environmental exposure to amines (9).

Concerning diagnostic methods, ultrasound is generally the first choice. Besides its non-invasive nature, it is specific, with no false positives (3). The use of CT scan should be balanced in young patient because of the costs and the risks of radiation exposure (3). MRI could be used as a diagnostic tool (7). In our case, ultrasound findings were confirmed by CT, but the definitive answer regarding extravesical extension was possible with MRI. Cystoscopy allows definitive diagnosis and staging (3) and, in many cases, also allows treatment (3, 6), as it was in the presented case.

Histopathological classification of UC, according to the 2004 World Health Organization/International Society of Urological Pathology (WHO/ISUP) criteria: urothelial papilloma (UP), papillary urothelial neoplasm of low malignant potential (PUNLMP), low-grade papillary urothelial carcinoma (LGPUC), and high-grade papillary urothelial carcinoma (HGPUC). Most of the small pediatric series describe these tumors as being characteristically low grade (5, 7, 8). Patients with urothelial papilloma have a low incidence of recurrence and rarely progress to develop urothelial carcinoma (10). LGPUC can contain a high-grade component and a lesion can be classified as a LGPUC even if the high-grade component comprises <5% (11). Our patient's first histopathological exam was consistent with urothelial papilloma. However, the lesion he presented 1 year later was classified as a LGPUC. A doubt remains if the later lesion was a progression of the disease to a LGPUC (more probable) or if the first biopsy had insufficient material to detect LGPUC.

The treatment of choice for bladder UC is transurethral resection of the bladder (TURB), with good results and low recurrence rate (4). Another option is to do an open resection, but this is reserved for patients with high-grade lesions. For our patient we elected TURB, and so far he has no recurrence after two and a half years of follow-up.

Currently there is no defined protocol for follow-up for pediatric UC. Some authors affirm that cystoscopy is the best diagnostic tool for detecting any recurrence (7). On the other hand, the low recurrence rate in papilloma and low grade tumors may allow following these patients with urinary cytology and bladder ultrasound alone (6), although urinary cytology has low-sensibility in detecting low-grade lesions in pediatric patients (5). The intensity of follow-up should be proportional to the risk of disease recurrence or progression (3).

Regarding the prognosis, most reported cases of bladder UC in pediatric population show a low grade malignancy and suggest an excellent prognosis with low recurrence and progression rates (2, 5). Two and a half years after surgery, our patient shows no recurrence. Nevertheless, follow-up for these patients should be mandatory, as recurrences can be multiple and can occur many years after the initial diagnosis, sometimes up to seven and a half years (4, 5).

Very few authors have published about the molecular and genetic profile of pediatric UC, therefore the analysis we performed in our patient is of great importance and can help to shed some light in a yet obscure subject. In adults, the most common molecular alterations in bladder UC includes fibroblast growth factor receptor 3 (FGFR3) and PI3K, which are associated with non-muscle-invasive papillary tumors; and mutations of tumor suppressor genes including p53, RB, and PTEN associated with invasive disease (12). Castillo-Martin et al. showed in a series of three pediatric TCC that there was no mutation in p53 and all three tumors had a H-RAS mutation, suggesting the existence of a different molecular pathway of bladder cancer tumorigenesis among pediatric population (12). In our analysis, we studied the KRAS gene—which was probably pathogenic. KRAS mutation are rare in urothelial carcinoma, but seems to be frequent in urachal carcinoma (13) and has already been described in inverted urothelial papilloma, urothelial papilloma, and advanced stage high-grade urothelial carcinoma in adult population (14, 15).

Also, in our analysis, patient's tumor presented a likely pathogenic variant in the BRAF gene. Recently, activating mutations in the BRAF gene, an important activator of MAP pathway, have been described in several tumor types including melanoma, colorectal, and papillary thyroid cancer. One study found BRAF mutation in 18% of urachal carcinoma, which frequency seems to be similar to that of in colorectal adenocarcinomas (13). Although Boulalas et al. studied the BRAF gene mutation in 30 patients with UC and found only in 2 patients this mutation (16).

There are only a few cases described in literature of pediatric bladder UC, and in a small minority of the described cases there is availability of tumor molecular and/or genetic profile, as presented in this paper. Besides that, further genetics studies need to be performed to assess the importance of KRAS and BRAF mutations in this disease.

## Data Availability Statement

This manuscript contains previously unpublished data. The name of the repository and accession number are not available.

## Ethics Statement

This study was carried out in accordance with the recommendations of Human Research Ethics Committee of the Clinical Hospital- Federal University of Paraná; with written informed consent from all subjects. All subjects gave written informed consent in accordance with the Declaration of Helsinki. The protocol was approved by the Human Research Ethics Committee of the Clinical Hospital- Federal University of Paraná.

## Author Contributions

MO and DS made study design, data collection, data analysis and interpretation, and manuscript writing. AF, LO, and BF were part of the surgical team and made data collection, data analysis, and interpretation. AA was the surgeon that performed the surgery and did data collection, data analysis, and interpretation. MA did revision of the manuscript. AD made critical revisions and approved final version. NA made English and grammar corrections, critical revisions, and approved final version. GL performed the tumor genetic profile analysis. CF did study design, data analysis and interpretation, manuscript writing, critical revisions, and approved final version.

### Conflict of Interest

The authors declare that the research was conducted in the absence of any commercial or financial relationships that could be construed as a potential conflict of interest.

## Abbreviations

UC: urothelial carcinoma
TURB: transurethral resection of bladder
TCC: transitional cell carcinoma
IRB: Institutional Review Board
CT: computed tomography
MRI: magnetic resonance imaging
WHO/ISUP: World Health Organization/International Society of Urological Pathology
UP: urothelial papilloma
PUNLMP: papillary urothelial neoplasm of low malignant potential
LGPUC: low-grade papillary urothelial carcinoma
HGPUC: high-grade papillary urothelial carcinoma
SEER: Surveillance Epidemiology and End Results.
